# Using Routinely Collected Hospital Data for Child Maltreatment Surveillance: Issues, Methods and Patterns

**DOI:** 10.1186/1471-2458-11-7

**Published:** 2011-01-05

**Authors:** Kirsten McKenzie, Debbie A Scott

**Affiliations:** 1 National Centre for Health Information Research and Training, School of Public Health, Queensland University of Technology, Victoria Park Road, Kelvin Grove QLD 4059, Australia

## Abstract

**Background:**

International data on child maltreatment are largely derived from child protection agencies, and predominantly report only substantiated cases of child maltreatment. This approach underestimates the incidence of maltreatment and makes inter-jurisdictional comparisons difficult. There has been a growing recognition of the importance of health professionals in identifying, documenting and reporting suspected child maltreatment. This study aimed to describe the issues around case identification using coded morbidity data, outline methods for selecting and grouping relevant codes, and illustrate patterns of maltreatment identified.

**Methods:**

A comprehensive review of the ICD-10-AM classification system was undertaken, including review of index terms, a free text search of tabular volumes, and a review of coding standards pertaining to child maltreatment coding. Identified codes were further categorised into maltreatment types including physical abuse, sexual abuse, emotional or psychological abuse, and neglect. Using these code groupings, one year of Australian hospitalisation data for children under 18 years of age was examined to quantify the proportion of patients identified and to explore the characteristics of cases assigned maltreatment-related codes.

**Results:**

Less than 0.5% of children hospitalised in Australia between 2005 and 2006 had a maltreatment code assigned, almost 4% of children with a principal diagnosis of a mental and behavioural disorder and over 1% of children with an injury or poisoning as the principal diagnosis had a maltreatment code assigned. The patterns of children assigned with definitive T74 codes varied by sex and age group. For males selected as having a maltreatment-related presentation, physical abuse was most commonly coded (62.6% of maltreatment cases) while for females selected as having a maltreatment-related presentation, sexual abuse was the most commonly assigned form of maltreatment (52.9% of maltreatment cases).

**Conclusion:**

This study has demonstrated that hospital data could provide valuable information for routine monitoring and surveillance of child maltreatment, even in the absence of population-based linked data sources. With national and international calls for a public health response to child maltreatment, better understanding of, investment in and utilisation of our core national routinely collected data sources will enhance the evidence-base needed to support an appropriate response to children at risk.

## Background

In Australia and many other countries, data on child maltreatment are largely derived from child protection agencies, predominantly reporting on events reported for investigation and/or substantiated cases of child maltreatment [1]. This approach underestimates the incidence of maltreatment [2,3] and makes inter-jurisdictional comparisons difficult, as each jurisdiction can have their own definition of what constitutes child maltreatment, unique definitions on what constitutes a report, and different processes for investigating and substantiating or refuting such reports. The World Health Organisation (WHO) in collaboration with the International Society for Prevention of Child Abuse and Neglect (ISPCAN) has called for common conceptual and operational definitions of child maltreatment to enable identification of cases across sectors involved in child maltreatment response and prevention [4]WHO and ISPCAN define child maltreatment as "all forms of physical and/or emotional ill-treatment, sexual abuse, neglect or negligent treatment or commercial or other exploitation, resulting in actual or potential harm to the child's health, survival, development or dignity in the context of a relationship of responsibility, trust or power" ([4], pg 9), with the four types grouped as physical abuse, sexual abuse, emotional or psychological abuse, and neglect.

Nationally and internationally, there has been a growing recognition of the importance of health professionals in identifying, documenting and reporting suspected child maltreatment [5,6] and for child maltreatment to be recognised as a public health problem [7]. The WHO in collaboration with the United Nations Children's Fund (UNICEF) called for child maltreatment to be recognised as a global public health concern, with an urgent recommendation for uniform reporting procedures to register both fatal and non-fatal child maltreatment [5]. The WHO highlighted health professionals as being in the best position of all professions to obtain evidence of child maltreatment, and called for better systems to enable communication between health professionals and social services.

If health professionals identified, documented and reported suspected child maltreatment routinely in patients medical records, statistical data derived from these records could potentially provide information about populations at risk as well as populations where maltreatment is already present (as provided by data derived from child protection agencies). To use hospital morbidity data for surveillance of child maltreatment, clear operational case definitions are required to ensure the system is both sensitive and specific to ensure accurate identification of true cases and limited inclusion of false cases [4].

The information contained in the medical record is coded according to the International Classification of Diseases and Related Health Problems (ICD) system developed by the WHO [8], which provides an internationally standardised system for classifying and aggregating diseases, injuries, causes of injuries and related health conditions for statistical purposes. To provide greater specificity for morbidity data collection, many countries modify the international ICD for clinical purposes. The WHO sanctions these clinical versions, with all of these versions of the classification required to be comparable to the international version at the three/four character code level, though supplemental characters may be added to the base three/four character code to increase specificity for clinical purposes. In Australia as well as many other countries, including New Zealand, Ireland, Germany, Romania, Slovenia and Saudi Arabia[8], the clinical modification that is used is the ICD-10 Australian Modification (ICD-10-AM) ([9].

As the ICD is a statistical classification, with specificity of case identification a critical element, the ICD provides stringent rules which coders are required to follow when assigning ICD codes to medical records. For a clinical coder to apply a definitive maltreatment code ('T74 Maltreatment syndromes', 'Y06 Neglect and abandonment' or 'Y07 Other maltreatment syndromes'), there must be clear clinical documentation of evidence of maltreatment. If the presentation is injury related, the coder is first directed to assign a code/s to describe the nature of the injury itself or if the presentation is disease related, the nature of the disease and then a code/s to describe the external cause of the injury. A single external cause code includes three dimensions: intent (i.e. accidental, assault, or undetermined), mechanism (i.e. struck by, fall/push, poisoning etc), and object/substance involved (i.e. knife, firearm, etc)). If documentation in the medical record indicates that the cause of the injury/disease is 'queried' or 'suspicious' of maltreatment but evidence of further investigation to rule out maltreatment or to substantiate it is not documented, the coder cannot assign a definitive maltreatment code. Instead the coder may assign a range of codes indicating possible maltreatment (such as 'Z04.4 Examination and observation following alleged rape and seduction') or problems related to previous alleged maltreatment ('Z61.4 Problems related to alleged sexual abuse of child by person within primary support group') [9].

An outcome of these rigid coding requirements is that case identification using morbidity data is likely to be specific (i.e. limiting inclusion of false cases), but not sensitive (i.e. not identifying all true cases). Hence morbidity data are likely to underestimate the true magnitude of child maltreatment if the clinical documentation is not clear, concise and complete and unless a broad range of indicative codes are used to identify possible cases of child maltreatment. Schnitzer et al found that ICD-9-CM codes were highly specific indicators of maltreatment, with 100% of records containing documentation about maltreatment, almost 90% regarding current maltreatment and just over 10% describing a history of maltreatment [10]. However, Winn et al found 25% of injuries to children resulting from violence identified through a multi-hospital surveillance system were not coded as assault-related using ICD external cause codes and concluded that assault-related external cause codes were specific (99.7% specificity) but not sensitive (74.6% sensitivity) [11]. Recognising that definitive maltreatment codes are likely to miss true maltreatment cases, a variety of diagnosis and external cause ICD codes have been used in an attempt to better identify cases of child maltreatment in health data sets [12]. Recent research conducted using linked hospital data and child protection data for a prospective case-control birth cohort found risks of allegations of child maltreatment and risks of substantiations of child maltreatment increased by 1.49 and 1.74 respectively for each single hospital admission per year [13]. Furthermore, cases with a discharge diagnosis of a mental or behavioural disorder had a 26 times greater risk of a child maltreatment substantiation and cases with a discharge diagnosis of an injury or poisoning had a 21 times greater risk of a child maltreatment substantiation compared to other discharge diagnoses [13].

Even in the absence of population-based linked databases, hospital morbidity data collections could enable an efficient population-based nationally standardised source of data to provide information on child maltreatment-related separations and at-risk populations. However, this would rely on clear documentation and more inclusive coding to enable the coding of both cases of queried as well as confirmed maltreatment (whilst still clearly differentiation such cases). However, to date, there has been no research conducted which explores the issues, methods and patterns of child maltreatment information in routinely collected health data collections in the Australian context and only limited research, using previous versions of the classification system, at an international level. The aim of this paper is to stimulate discussion and contribute to the future development of non-fatal child maltreatment surveillance initiatives. This study aimed to describe the issues around case identification using ICD coded morbidity data, outline methods for selecting and grouping relevant codes, and illustrate patterns of maltreatment identified when using the outlined approach.

## Methods

This study involved a systematic review of the ICD-10-AM classification system underpinning hospitalisation data to identify relevant codes for case identification of maltreatment-related presentations and a descriptive analysis of these data to explore patterns of code utilisation.

### Classification system review

A comprehensive review of the ICD-10-AM classification system was undertaken to identify the range of possible codes to be used for identification of maltreatment-related presentations, and to review the guidelines and rules affecting the use of these codes. To identify the range of ICD-10-AM codes, a search was conducted of all ICD-10-AM index terms for diseases, external causes, and procedures referring to abuse (excluding the phrase 'substance abuse') or maltreatment. Additionally, a free text search of ICD-10-AM 6th edition tabular volumes was performed to search for 'abuse' (excluding the phrase 'substance abuse') and 'maltreatment' to ensure there were no additional codes which were missed in the index search. Coding standards in the ICD-10-AM pertaining to child maltreatment coding were also reviewed in order to understand the instructions provided to coders regarding the appropriate assignment of codes around child maltreatment [8]. The relevant coding standards which were identified were 1909 Adult and Child Abuse, 2008 Perpetrator of Assault, Abuse and Neglect, and 0526 Münchhausen's by Proxy.

Potential flags for maltreatment-related events included both codes that signified a current episode of maltreatment and codes which signified a prior history of maltreatment. The rationale for this approach was that for any maltreatment codes (current or prior) to be coded as co-morbidities in the medical records the patient needed to have been treated for and/or had their hospital stay extended due to the condition which was coded. Codes were only included if they indicated specific reference to maltreatment in order to avoid capturing a large number of broad conditions through non-specific codes (such as 'Z61.8 Other negative life events in childhood', 'Z61.9 Negative life event in childhood unspecified', 'Z62.8 Other specified problems related to upbringing', 'Z62.9 Problem related to upbringing unspecified' etc. 'F94.1 Reactive attachment disorder of childhood' which is defined as "a disorder starting in the first five years of life with abnormal patterns of social relationships, probably as a direct result of severe abuse or neglect" was excluded as it specifically excludes maltreatment syndromes (T74) and sexual or physical abuse in childhood resulting in psychosocial problems (Z61.4-Z61.6).

In ICD-10-AM, fifth character supplemental codes are used to specify the perpetrator where cases are coded as being assault-related. The key perpetrator codes for identification of maltreatment include the specific perpetrator codes for Parent, Other family member, Carer, as well as the non-specific codes for 'Other specified person' and 'Unspecified person' (as these codes may be used where there isn't an appropriate code in the classification system to capture the perpetrator, or where the documentation doesn't provide information about the perpetrator). For children 14 years of age or younger, cases were included in the 'maltreatment' category if either a specific perpetrator code for Parent, Other family member, Carer was used or a non-specific code for 'Other specified person' and 'Unspecified person' was used. For children 15 to 17 years of age, cases were only included in the 'maltreatment' category if a specific perpetrator code for Parent, Other family member, Carer was used. This restriction was applied as early exploratory analysis demonstrated that a large portion of assault cases for teenagers between the age of 15 and 18 were coded as being caused by an 'other specified' perpetrator (n = 70 out of a total of 1615 cases which had a perpetrator code) or 'unspecified' perpetrator (n = 989 out of a total of 1615 cases which had a perpetrator code). It is likely that the majority of these cases were due to peers/partners and as these cases were not in the scope of the definition of maltreatment used in this study, these cases were not included in this analysis to ensure a conservative estimate of maltreatment-related cases.

Following the selection of potential codes, expert health information managers were consulted to review the list, ensuring broad coverage of maltreatment codes within the classification system. In addition, two separate consultations were conducted with emergency department (ED) clinicians at two major paediatric hospitals to identify any other additional codes that the review process may have overlooked. Following consultation with ED clinicians the procedure code for 'skeletal survey' was included, as this was acknowledged to be a key diagnostic procedure conducted when child maltreatment was suspected. The final list of codes used for identification of cases of maltreatment included diagnosis codes, external cause codes and procedure codes and these are shown in Table 1.

**Table 1:** ICD Codes Selected for Case Identification of Maltreatment-Related Cases

Type of Code	ICD-10-AM Code	ICD-10-AM Code Description
Diagnosis Codes	T74.0	Maltreatment syndromes: Neglect or abandonment
	T74.1	Maltreatment syndromes: Physical Abuse
	T74.2	Maltreatment syndromes: Sexual abuse
	T74.3	Maltreatment syndromes: Psychological abuse
	T74.8	Maltreatment syndromes: Other maltreatment syndromes
	T74.9	Maltreatment syndromes: Maltreatment syndrome, unspecified
	Z04.4	Examination and observation following alleged rape and seduction
	Z04.5	Examination and observation following other inflicted injury
	Z61.4	Problems related to alleged sexual abuse of child by person within primary support group
	Z61.5	Problems related to alleged sexual abuse of child by person outside primary support group
	Z61.6	Problems related to alleged physical abuse of child
	Z62.0	Inadequate parental supervision and control
	Z62.3	Hostility towards and scapegoating of child
	Z62.4	Emotional neglect of child
	Z62.5	Other problems related to neglect in upbringing
	Z62.6	Inappropriate parental pressure and other abnormal qualities of upbringing

External Cause Codes	X85-Y09	Assault codes - includes 5th character perpetrator codes as follows:
	1	Parent
	2	Other family member
	3	Carer
	8	Other specified person*
	9	Unspecified person*

Procedure Codes	5830600	Radiography of whole skeleton
	9608400	Physical Abuse Counselling

*Cases where an 'other specified' or 'unspecified' perpetrator were only included in the maltreatment group if the child was 14 years of age or under (see methodology section for further explanation)

As the WHO categorises maltreatment into physical abuse, sexual abuse, emotional or psychological abuse, and neglect, ICD maltreatment codes were further grouped to create variables to flag the presence of each of these forms of maltreatment. Table 2 provides a summary of the code groupings used to create these variables. For cases with any maltreatment coded, descriptive analysis was used to show the frequency of each type of maltreatment and the number of maltreatment types assigned by patient demographics.

**Table 2:** ICD Code Groupings by Maltreatment Type

Maltreatment Type	ICD-10-AM Code	ICD-10-AM Code Description
Neglect	T74.0	Maltreatment syndromes: Neglect or abandonment
	Y06	Neglect and abandonment
	Z62.0	Inadequate parental supervision and control
	Z62.4	Emotional neglect of child
	Z62.5	Other problems related to neglect in upbringing

Physical Abuse	T74.1	Maltreatment syndromes: Physical Abuse
	X85-Y04, Y08-Y09	Assault (excluding 'Y05 Sexual assault', 'Y06 Neglect and abandonment', and 'Y07 Other maltreatment syndromes')
	Z04.5	Examination and observation following other inflicted injury
	Z61.6	Problems related to alleged physical abuse of child

Sexual Abuse	T74.2	Maltreatment syndromes: Sexual abuse
	Y05	Sexual assault by bodily force
	Z04.4	Examination and observation following alleged rape and seduction
	Z61.4	Problems related to alleged sexual abuse of child by person within primary support group
	Z61.5	Problems related to alleged sexual abuse of child by person outside primary support group

Emotional or Psychological Abuse	T74.3	Maltreatment syndromes: Psychological abuse
	Z62.3	Hostility towards and scapegoating of child
	Z62.6	Inappropriate parental pressure and other abnormal qualities of upbringing

Other or Unspecified	T74.8	Maltreatment syndromes: Other maltreatment syndromes
	T74.9	Maltreatment syndromes: Maltreatment syndrome, unspecified
	Y07	Other maltreatment syndromes

### Statistical Analysis

Descriptive analysis of National Hospital Morbidity Database (NHMD) was conducted for children under 18 years of age (to comply with the operational definition of 'child' in the *Child Protection Act *(1999) which governs Queensland child protection [14]) who were admitted to an Australian hospital between 1 July 2005 and 30 June 2006. The database is collated by the Australian Institute for Health and Welfare (AIHW) using summary records for hospital discharges provided by state and territory health authorities and contains data from 1 July 1993 to 30 June 2008. The AIHW technical appendices of the national hospital statistics reports suggest the quality of the data contained in this database are relatively good and this database is routinely used by the AIHW and other key health agencies to provide national morbidity statistics [15].

PASW Version 18 was used to conduct descriptive analyses, using frequencies and percentages to quantify the numbers and proportions of patients assigned relevant child maltreatment related codes and to explore the characteristics of cases assigned any child maltreatment related codes. Ethics approval to conduct this analysis was obtained from the relevant Human Research Ethics Committees.

## Results

### National hospitalisation separation data for cases with and without a maltreatment code

There were 647,819 hospital separations for children under the age of 18 years in Australia during the financial year 2005-2006. Exploratory analyses found that, of the 647,819 total separations, 2120 (0.3%) had a maltreatment code present in their hospitalisation data (See Table 3). While males comprised the larger proportion of hospital separations overall (55.4%), females comprised the larger proportion of hospital separations where a maltreatment code was assigned (57.8%). For males the age group with the highest proportion of cases with a maltreatment code assigned was the 10-14 year olds with 0.5% of the total hospital separations having a maltreatment code assigned, compared to females where the 15-17 year old age group for females had the highest proportion of maltreatment codes assigned (0.9% of total hospital separations for females).

**Table 3:** Age Group and Gender and Presence of Maltreatment Code of Australian Children Hospitalised between 1 July 2005 and 30 June 2006

Sex and Age Groups	Any Maltreatment Code		No Maltreatment Code		Total
	n	%	n	%	n
Males					
< 1	180	0.2	79982	99.8	80162
1-5	160	0.1	115821	99.9	115981
6-9	85	0.2	52370	99.8	52455
10-14	327	0.5	60590	99.5	60917
15-17	143	0.3	49602	99.7	49745
Total	895	0.2	358365	99.8	359260

Females					
< 1	132	0.2	59032	99.8	59164
1-5	177	0.2	82337	99.8	82514
6-9	45	0.1	38154	99.9	38199
10-14	337	0.7	47252	99.3	47589
15-17	534	0.9	60541	99.1	61075
Total	1225	0.4	287316	99.6	288541

Total					
< 1	312	0.2	139029	99.8	139341
1-5	337	0.2	198160	99.8	198497
6-9	130	0.1	90525	99.9	90655
10-14	664	0.6	107842	99.4	108506
15-17	677	0.6	110143	99.4	110820

Total (missing n = 18)	2120	0.3	645699	99.7	647819

To examine the broad principal diagnoses of children with any maltreatment code present compared to those without a maltreatment code present, analysis was conducted comparing these group by ICD chapter of the principal diagnosis(See Table 4). Separations with a principal diagnosis from the mental and behavioural disorders chapter had the highest proportion of cases where a maltreatment code was assigned accounting for 3.6% of cases. Separations with a principal diagnosis from the injury and poisoning chapter had the second highest proportion of cases where a maltreatment code was assigned accounting for 1.1% of cases. Furthermore, of all cases with a maltreatment code assigned, the injury and poisoning chapter was the most common principal diagnosis chapter, accounting for 46.3% of cases where a maltreatment code was assigned.

**Table 4:** ICD-10-AM Chapter for Principal Diagnosis for children with maltreatment code present in Diagnosis String in Australia July 2005-June 2006

	Any Maltreatment Coded		No Maltreatment Coded		Total
**ICD-10-AM Chapters for Principal Diagnosis**	**n**	**%**	**n**	**%**	**n**

Infectious and Parasitic	27	0.1	43630	99.9	43657
Neoplasms	5	0.0	14988	99.9	14993
Blood, Blood-Forming and Immune System	2	0.0	8615	99.9	8617
Endocrine, Nutritional and Metabolic	13	0.1	9381	99.9	9394
Mental and Behavioural	652	3.6	17263	96.4	17915
Nervous System	16	0.1	21254	99.9	21270
Eye and Adnexa	6	0.1	6373	99.9	6379
Ear and Mastoid Process	5	0.0	29157	99.9	29162
Circulatory	2	0.0	4421	99.9	4423
Respiratory	37	0.0	101199	99.9	101236
Digestive	15	0.0	74143	99.9	74158
Skin and Subcutaneous Tissue	28	0.2	17153	99.8	17181
Musculoskeletal and Connective Tissue	8	0.0	16211	99.9	16219
Genitourinary	6	0.0	19582	99.9	19588
Pregnancy, Childbirth, and Puerperium	22	0.2	9670	99.8	9692
Perinatal	22	0.0	54033	99.9	54055
Congenital and chromosomal	9	0.0	23410	99.9	23419
Symptoms and signs NEC	64	0.1	43729	99.9	43793
Injuries and Poisonings	982	1.1	87982	98.9	88964
Factors Influencing Health Status	198	0.5	43182	99.5	43380

Total (missing n = 1)	2119	0.3	645376	99.7	647495

There were 2670 cases with a perpetrator code assigned in the dataset, and of these 1059 were excluded from inclusion in the maltreatment-related sample as they were coded as 'other' or 'unspecified' perpetrators for 15-17 year olds (as described in methods). For cases where a maltreatment code and a perpetrator code was assigned:

• Under 1 year of age: 188 cases had a parent, carer or family member coded as the perpetrator, 3 cases had a spouse coded, 44 cases had an 'other' or 'unspecified' perpetrator;

• 1-5 years: 166 cases had a parent, carer or family member coded, 3 cases had a spouse coded, and 55 cases had an 'other' or 'unspecified' perpetrator;

• 6-9 years: 52 cases had a parent, carer or family member coded, 1 case had a spouse coded, and 30 cases had an 'other' or 'unspecified' perpetrator;

• 10-14 years: 122 cases had a parent, carer or family member coded, 1 case had a spouse coded, and 145 cases had an 'other' or 'unspecified' perpetrator;

• 15-17 years: 122 cases had a parent, carer or family member coded, 10 case had a spouse coded as the perpetrator, and 38 cases had an 'other' or 'unspecified' perpetrator.

### Principal diagnosis for cases where a maltreatment code was assigned

Table 5 depicts the top five most commonly assigned ICD Principal Diagnosis codes (to the 3 character ICD-10-AM code level) per age group in those children who also had any of the relevant maltreatment codes in their separation data. For each age group, these top 5 principal diagnoses accounted for between one-third to one-half of all diagnoses in each age group.

**Table 5:** Top 5 Principal Diagnoses for Cases with an Maltreatment Code by Age Group

ICD-10-AM Principal Diagnosis Code by Age Group	n	%
< 1 year (n = 312)		
T74 Maltreatment syndromes	53	17.0
S00 Superficial injury of head	28	9.0
S06 Intracranial injury	20	6.4
S02 Fracture of skull and facial bones	17	5.4
S09 Other and unspecified injuries of head	11	3.5
S42 Fracture of shoulder and upper arm	11	3.5
S72 Fracture of femur	11	3.5
Remainder of age group	161	51.6

1-5 years (n = 337)		
T74 Maltreatment syndromes	37	11.0
S00 Superficial injury of head	32	9.5
Z04 Examination and observation for other reasons	29	8.6
Z61 Problems related to negative life event in childhood	17	5.0
S06 Intracranial injury	12	3.6
Remainder of age group	210	62.3

6-9 years (n = 130)		
F91 Conduct disorders	17	13.1
T74 Maltreatment syndromes	13	10.0
S00 Superficial injury of head	7	5.4
S09 Other and unspecified injuries of head	7	5.4
S05 Injury of eye and orbit	5	3.8
Remainder of age group	81	62.3

10-14 years (n = 664, missing n = 1)		
F43 Reaction to severe stress, and adjustment disorders	75	11.3
S02 Fracture of skull and facial bones	63	9.5
F32 Depressive episode	32	4.8
S00 Superficial injury of head	32	4.8
S09 Other and unspecified injuries of head	28	4.2
Remainder of age group	433	65.3

15-17 years (n = 677)		
F43 Reaction to severe stress, and adjustment disorders	141	20.8
F32 Depressive episode	69	10.2
F33 Recurrent Depressive disorder	62	9.2
F60 Specific personality disorders	31	4.6
Z04 Examination and observation for other reasons	28	4.1
Remainder of age group	346	51.1

Total	2119	100

T74 (Maltreatment Syndromes) was the most commonly assigned principal diagnosis code for children under 5 years of age. Where injury codes were assigned as the principal diagnosis, head injuries were the most common injury coded for all children under 15 years of age. For the 6-9 year olds, the most common principal diagnosis for cases coded with a maltreatment code was 'F91 Conduct disorder', accounting for 13% of separations in this age group. In the 10-14 year old age group a mixture of injury and mental and behavioural disorder codes were present as the top principal diagnoses. In the oldest group of children, those from 15-17 years of age, the majority of Principal Diagnosis codes assigned were associated with Mental and Behavioural Disorders with the top 4 principal diagnoses in this chapter accounting for 45% of separations in this age group.

### Most common maltreatment types assigned by age group and sex

Table 6 shows the pattern of maltreatment types by age group and sex (Note: each child could have more than one maltreatment code assigned reflecting different maltreatment types). For males physical abuse was most commonly coded (62.6%). This was true for all age groups except children under the age of one year where the largest proportion (48.3%) was assigned an 'Other abuse' code. In females with a maltreatment code, sexual abuse was the most commonly assigned form of maltreatment accounting for 52.9% of cases. When analysed by age group however, this was only true for those females older than 10 years of age with 59.3% of girls aged 10-14 and 73.4% of girls aged 15-17 with a maltreatment code being assigned a 'Sexual Abuse' code. In females younger than 1 year of age, the largest proportion were assigned an 'Other abuse' code (37.9%), and in females aged 1-5 and 6-9 females, 'Physical Abuse' was the most common maltreatment type assigned (36.2% and 55.6% respectively).

**Table 6:** Age group and sex by maltreatment type

Sex and Age Groups	Neglect		Physical		Sexual		Psychological		Other/Unspec	
	n	% present	n	% present	n	% present	n	% present	n	% present
Males										
<1	46	25.6	62	34.4	2	1.1	2	1.1	87	48.3
1-5	50	31.3	66	41.3	7	4.4	2	1.3	50	31.3
6-9	17	20.0	60	70.6	7	8.2	2	2.4	11	12.9
10-14	28	8.6	277	84.7	29	8.9	3	.9	18	5.5
15-17	17	11.9	95	66.4	37	25.9	1	.7	3	2.1
Total	158	17.7	560	62.6	82	9.2	10	1.1	169	18.9

Females										
< 1	47	35.6	49	37.1	2	1.5	1	.8	50	37.9
1-5	42	23.7	64	36.2	44	24.9	1	.6	49	27.7
6-9	11	24.4	25	55.6	10	22.2	0	.0	7	15.6
10-14	27	8.0	139	41.2	200	59.3	3	.9	25	7.4
15-17	44	8.2	131	24.5	392	73.4	8	1.5	25	4.7
Total	171	14.0	408	33.3	648	52.9	13	1.1	156	12.7

Total										
< 1	93	29.8	111	35.6	4	1.3	3	1.0	137	43.9
1-5	92	27.3	130	38.6	51	15.1	3	.9	99	29.4
6-9	28	21.5	85	65.4	17	13.1	2	1.5	18	13.8
10-14	55	8.3	416	62.7	229	34.5	6	.9	43	6.5
15-17	61	9.0	226	33.4	429	63.4	9	1.3	28	4.1

Total	329	15.5	968	45.7	730	34.4	23	1.1	325	15.3

Most children, regardless of age or gender had only one type of maltreatment coded. In males only 3% of cases had more than one type of maltreatment coded, with most of these aged over 10 years of age (the age breakdown of males where more than one type of maltreatment was coded was: <1 yr n = 2, 6-9 yrs n = 5, 10-14 yrs n = 15, and 15-17 yrs n = 7). In females only 6% of cases had more than one type of maltreatment coded, with most of these aged over 10 years of age (the age breakdown of females where more than one type of maltreatment was coded was: <1 yr n = 2, 1-5 yrs n = 3, 6-9 yrs n = 1, 10-14 yrs n = 35, and 15-17 yrs n = 31).

## Discussion

This study undertook a review of routinely collected and coded hospital data as a source of information on child maltreatment. While less than 0.5% of children overall hospitalised in Australia between 2005 and 2006 had a maltreatment code assigned in their separation data, almost 4% of children with a principal diagnosis of a mental and behavioural disorder and over 1% of children with an injury or poisoning as the principal diagnosis had a maltreatment code assigned. This result supports previous research which identified that cases with a discharge diagnosis of a mental or behavioural disorder or an injury or poisoning had significantly increased risk of child maltreatment substantiation using linked hospital and child protection data [13]^.^

While the ICD-10-AM classification system provides both definitive codes for child maltreatment (such as T74 Maltreatment Syndrome) as well as possible codes for maltreatment (such as those indicating examination for or problems related to previous alleged maltreatment), in the current data, only 18.8% of cases identified as having any of the range of maltreatment codes were coded using the definitive T74 Maltreatment Syndrome code. Relying on this code alone would have significantly underestimated the prevalence of maltreatment-related presentations, with previous research indicating that definitive maltreatment codes are highly specific but not sensitive indicators of maltreatment [10,11]. The codes more commonly assigned for those cases identified as having a maltreatment code were those referring to problems related to the *alleged *sexual abuse (27.4% Z61.4 and Z61.5) or *alleged *physical abuse (10.8% Z61.6).

The most commonly assigned principal diagnosis code in those children coded with a maltreatment code varied according to age group. In those younger than 5 years, the 'T74 Maltreatment syndrome' case was the most commonly assigned principal diagnosis code for children under 5 years of age', similar to O'Donnell et al who found that most children with a maltreatment code were aged under 5 years [16]. For older children the most common principal diagnosis for 6-9 year olds was 'F91 Conduct disorder', in the 10-14 year old age group a mixture of injury and mental and behavioural disorder codes, and for 15-17 year olds were also mental and behavioural disorders. A higher proportion of mental and behavioural disorder codes and injury codes for children who are known to the child protection system has previously been identified by researchers in Western Australia [13]. Patterns of maltreatment types varied by sex and age group. Physical abuse was most common overall for males with 62.6% of males with a maltreatment code assigned a physical abuse code. Sexual abuse was most commonly assigned overall for females with 52.9% of females with a maltreatment code assigned a sexual abuse code. Similarly, O'Donnell et al found a much higher proportion of females (71.4%) than males (28.6%) aged between 2-13 years with a notified sexually transmitted infection [16]. However, in females younger than 1 year of age, the largest proportion of cases with a maltreatment code were assigned an 'Other abuse' code (37.9%), and in females aged 1-5 and 6-9 females, the largest proportion of cases were assigned a 'Physical Abuse' code (36.2% and 55.6% respectively).

For cases with a maltreatment code and a perpetrator code, the most common perpetrator coded for all age groups up to 9 years was parent, carer or family member. However, within these age groups there were 7 cases coded as having a perpetrator of 'spouse', which is defined as the spouse of the injured patient. It is possible that these cases were cases of child maltreatment by the 'spouse' of the parent (i.e. step-parent or defacto partner of parent) which were wrongly assigned a 'spouse' code. A conservative estimate of maltreatment-relatedness of cases was used in this study, through removing all other and unspecified perpetrator codes for children aged 15-17 years of age. This may have underestimated the number of cases in this age group who were victims of child maltreatment, and further research is needed to examine this group in more detail to enable a better estimate of the likely proportion of child maltreatment cases for 15-17 year olds. However, recent research has found that around 50% of interpersonal violence cases (all ages) are assigned an 'other' or 'unspecified' perpetrator code [17].

Patterns of diagnoses by maltreatment codes/maltreatment types can provide valuable insight into the epidemiology of severe child maltreatment and the resultant harms to children over time. Were researchers able to have some confidence in the sensitivity and specificity of maltreatment codes, these data could form a valuable source of information to target intervention, reduction and prevention initiatives.

A potential limitation of this study, and any study reliant on the clinical coding of morbidity data, is the requirement for complete and accurate clinical documentation and consistent, quality clinical coding to ensure reliable coded data. The capture of complete, timely, relevant and accurate maltreatment data is reliant on a number of processes. Firstly there needs to be a suspicion of child maltreatment in the clinical context, the clinical documentation of indicators of maltreatment for children suspected of maltreatment needs to be provided and the coder needs to assign relevant ICD codes to identify the type of maltreatment documented. If the documentation in the medical record is ambiguous, incomplete or illegible, coders are unable to assign appropriate codes. This could result in underestimations of child maltreatment in coded morbidity data. In order to quantify this underestimation a medical record and coding audit is required, and further stages of our current research study seek to explore these issues.

## Conclusions

This study has demonstrated that hospital morbidity data could provide valuable information for routine monitoring and surveillance of non-fatal child maltreatment even in the absence of population-based linked data sources. ICD coded hospital morbidity data provides codes for both definitive and possible maltreatment as well as types of maltreatment, and being a statistical classification, there are strict rules and definitions to improve the specificity of case identification. Further work is needed to evaluate the reliability of coding of identified maltreatment codes and to enumerate the likely sensitivity and specificity of the system for case identification of maltreatment-related presentations. In the meantime, health professionals and administrators can use this information to conduct follow-up audits of possible cases of maltreatment. Where linked databases are available, researchers could use these codes to further interrogate hospitalisation data for indicators of maltreatment in addition to the use of disease diagnosis codes and child protection outcome data. With national and international calls for a public health response to child maltreatment, better understanding of, investment in and utilisation of our core national routinely collected data sources will enhance the evidence-base needed to support an appropriate response to children at risk.

## Competing interests

The authors declare that they have no competing interests.

## Authors' contributions

Both authors made an important contribution to the development of the ideas and conduct of the research. Both authors were primarily responsible for developing the first drafts of the manuscript; KM made a major contribution to the analysis. All authors have been integrally involved in reviewing and extensively editing the text as it has progressed through several iterations.

## Pre-publication history

The pre-publication history for this paper can be accessed here:

http://www.biomedcentral.com/1471-2458/11/7/prepub
